# Predictive value of circulating lymphocyte subsets and inflammatory indexes for neoadjuvant chemoradiotherapy response in rectal mucinous adenocarcinoma patients: A machine learning approach

**DOI:** 10.1002/cam4.7416

**Published:** 2024-07-24

**Authors:** Yu Lin, Yanwu Sun, Weizhong Jiang, Yu Deng, Ying Huang, Pan Chi

**Affiliations:** ^1^ Department of Colorectal Surgery Fujian Medical University Union Hospital Fuzhou Fujian People's Republic of China

**Keywords:** chemoradiotherapy, circulating lymphocyte, inflammatory indexes, machine learning, mucinous adenocarcinoma, rectal cancer

## Abstract

**Introduction:**

In this study, we aimed to evaluate the predictive value of circulating lymphocyte subsets and inflammatory indexes in response to neoadjuvant chemoradiotherapy (NCRT) in patients with rectal mucinous adenocarcinomas (MACs).

**Methods:**

Rectal MAC patients who underwent NCRT and curative resection at Fujian Medical University Union Hospital's Department of Colorectal Surgery between 2016 and 2020 were included in the study. Patients were categorized into good and poor response groups based on their pathological response to NCRT. An independent risk factor‐based nomogram model was constructed by utilizing multivariate logistic regression analysis. Additionally, the extreme gradient boosting (XGB) algorithm was applied to build a machine learning (ML)‐based predictive model. Feature importance was quantified using the Shapley additive explanations method.

**Results:**

Out of the 283 participants involved in this research, 190 (67.1%) experienced an unfavorable outcome. To identify the independent risk factors, logistic regression analysis was performed, considering variables such as tumor length, pretreatment clinical T stage, PNI, and Th/Tc ratio. Subsequently, a nomogram model was constructed, achieving a C‐index of 0.756. The ML model exhibited higher prediction accuracy than the nomogram model, achieving an AUROC of 0.824 in the training set and 0.762 in the tuning set. The top five important parameters of the ML model were identified as the Th/Tc ratio, neutrophil to lymphocyte, Th lymphocytes, Gross type, and T lymphocytes.

**Conclusion:**

Radiochemotherapy sensitivity is markedly influenced by systemic inflammation and lymphocyte‐mediated immune responses in rectal MAC patients. Our ML model integrating clinical characteristics, circulating lymphocyte subsets, and inflammatory indexes is a potential assessment tool that can provide a reference for individualized treatment for rectal MAC patients.

## 1 INTRODUCTION

Mucinous adenocarcinoma (MAC) is a distinct subset of rectal cancer with distinctive molecular characteristics and aggressive biological behavior. For patients with locally advanced rectal cancer, neoadjuvant chemoradiotherapy (NCRT) is frequently used as part of the multimodal treatment. Prior research has demonstrated a less favorable response to NCRT in rectal MAC patients compared with rectal non‐MAC patients.^1^ However, recent findings suggest that rectal MAC patients may also experience advantages from modern multimodality therapies.^2^ Therefore, accurately predicting the response to NCRT in rectal MAC patients would speed up personalized treatment strategies and reduce the instances of ineffective interventions.

New findings indicate that the effectiveness of NCRT treatment is affected by the biological properties of cancer cells, tumor microenvironment, and immune response of the host.^3^ Peripheral white blood cells and lymphocytes^4^, ^5^ can indicate systemic immune and inflammatory responses against tumors. In our previous study, we demonstrated that the initial levels of hematological inflammatory markers, such as the ratio of neutrophils to lymphocytes (NLR), ratio of platelets to lymphocytes (PLR), systemic immune–inflammation index (SII), and prognostic nutritional index (PNI), could accurately forecast the outcome of rectal MAC patients.

This finding underscores the significance of the above factors in predicting the prognosis of rectal MAC patients.^6^ In this study, we aimed to further investigate the predictive value of the circulating lymphocyte subpopulations in response to NCRT in rectal MAC patients. By developing a precise and user‐friendly prediction tool, we hope to enhance the accuracy and ease of treatment response prediction.

Machine learning (ML), a branch of artificial intelligence (AI) and computer science, aims to enhance accuracy by utilizing statistics and relevant algorithms to mimic human brain learning. ML‐based algorithms have demonstrated impressive capabilities in the areas of disease diagnosis, forecasting prognosis, assessing the effectiveness of antitumor drugs, and evaluating treatment response, among others.^7^ However, only a few studies have applied ML algorithms to evaluate tumor response in rectal MAC patients after NCRT.

The objective of this study in this situation was to create an ML model that combines hematological inflammatory indicators and lymphocyte subsets to forecast tumor response to NCRT in rectal MAC patients. This prediction could potentially offer guidance for personalized treatment of rectal MAC patients.

## 2 MATERIALS AND METHODS

### 2.1 Patients

We used our meticulously updated database to identify and analyze the data of patients with rectal MACs who were treated at Fujian Medical University Union Hospital's Department of Colorectal Surgery between 2016 and 2020. To be eligible for the study, patients had to meet the following requirements: histologically verified MAC, tumors located within 12 cm from the anal verge, and successful completion of NCRT followed by radical surgery. Exclusion criteria included distant metastasis at diagnosis, synchronous malignancy or a history of other malignant tumors, emergency surgery, palliative resection, local excision, or a “watch‐and‐wait” strategy, evidence of acute or chronic inflammatory conditions or hematologic diseases, and incomplete medical records. The flow diagram of the study population is presented in Supplementary Figure S1. The institutional review board of our hospital approved this retrospective study, and individual informed consent was waived.

### 2.2 Treatment

Tumor and nodal staging were determined based on a combination of digital rectal examination, colonoscopy, abdominopelvic MRI, and/or transrectal ultrasonography. Systemic staging required a chest CT scan. After evaluating the advantages and disadvantages of treatment options, the primary factor in determining the use of NCRT for rectal MAC patients was the stage of the tumor (cT3–4 and/or cN+). The pelvis received preoperative radiotherapy at a dosage of 45 Gy, followed by a 5–6‐week period for a primary tumor boost of 5.4 Gy. Concurrent chemotherapy was administered using single or double regimens, such as capecitabine, FOLFOX, or CAPOX. Surgical procedures, including low anterior resection and abdominoperineal resection, were carried out 6–8 weeks after the completion of radiotherapy. Two independent pathologists evaluated the tumor's response to NCRT based on the four‐tier classification of tumor regression grade (TRG) by the American Joint Committee on Cancer (AJCC). “Good responders” were defined as those with TRG 0–1, while “poor responders” were defined as those with TRG 2–3.^8^

### 2.3 Hematological inflammation‐based indexes and lymphocyte subsets

Blood samples from the peripheral veins were collected prior to the initiation of NCRT. Blood samples were obtained in collection tubes containing ethylene diamine tetra‐acetic acid, and the blood cell counts were analyzed using an automated hematology analyzer. Hematological inflammation‐based indexes were calculated using the following equations: albumin‐to‐globulin ratio (AGR) = albumin/(total serum protein − albumin) and PNI = serum albumin (g/L) + 5 × total lymphocyte count (10^9^/L). NLR = neutrophil count/lymphocyte count, PLR = platelet count/lymphocyte count, and SII = platelet count × neutrophil count/lymphocyte count. Lymphocyte subsets were identified by multicolor flow cytometry as follows: CD3^+^/CD19^−^ for T lymphocytes, CD3^+^/CD4^+^ for helper T lymphocytes (Th lymphocytes), CD3^+^/CD8^+^ for cytotoxic T lymphocytes (Tc lymphocytes), and CD3^−^/CD56^+^ for natural killer cells.

### 2.4 Machine learning model

We constructed and validated a forecast model for categorizing data in accordance with the guidelines of Transparent Reporting of a multivariable prediction model for Individual Prognosis Or Diagnosis (TRIPOD). A type 2A approach was adopted, which included dividing the sample randomly for both model development and validation.^9^ The individuals were separated into a training group, and a tuning group with a ratio of 7:3. Binary classifiers were constructed using the training set and subsequently utilized to predict the cluster labels on the tuning set. AI models were developed using the training set. To prevent data leakage, the Sklearn Standard Scaler was utilized on the training set and subsequently implemented on the training and tuning sets after scaling the data.

During the training procedure, a total of 26 features, encompassing clinical characteristics, laboratory tests, and imaging parameters that were accessible prior to NCRT, were utilized. To predict the reaction of tumors in patients with rectal MACs, the extreme gradient boosting (XGB) algorithm was applied.^10^ This algorithm iteratively constructs classification and regression trees, optimizes the loss function, and controls model complexity to avoid overfitting using regularization terms involving leaf nodes. The splitting of the decision tree continues until the model's specified minimum loss function and complexity threshold are achieved. In this study, the final prediction probabilities were calculated from all trees for an accurate prediction of treatment response. During the training phase, the model's hyperparameters were individually optimized for each tree in the training dataset through random sampling. Imbalanced data was corrected by undersampling and oversampling. To discover the best parameters and prevent overfitting of the data, a five‐fold cross‐validation technique was employed. The SHAP (SHapley Additive exPlanations) values, a game‐theoretic approach, allow for the quantification of the contribution of each feature to the prediction for every instance.^11^ This method is utilized to elucidate the individual contributions of each feature and the resulting output of the model.

### 2.5 Statistical analyses

Statistical analyses were performed using the IBM SPSS software (version 27 for Mac) and R software (version 3.6.2). Student's *t*‐test was utilized to analyze continuous data. When appropriate, a comparison of the categorized variables was conducted using either Fisher's exact test or the chi‐square test. A predictive nomogram was constructed based on the findings of the logistic regression analysis. The area under the ROC curve (AUROC) and Harrell's concordance index (C‐index) were calculated to evaluate the performance of the predictive model. The calibration of the nomogram was performed by comparing the predicted and actual probability after bias correction. The ML model was constructed utilizing the “xgboost,” “pROC,” “shapforxgboost,” “reshape2,” “ggplot2,” and “ggpubr” packages. The computational code for the algorithms can be found in the supplementary attachment (refer to xgboost.txt). A two‐sided *p* value <0.05 was regarded as statistically significant.

## 3 RESULTS

### 3.1 Patient characteristics

A total of 283 patients with rectal MACs were analyzed. Out of these patients, 93 patients (32.9%) were classified as the good response group for achieving a TRG of 0–1, whereas 190 patients (67.1%) were classified as the poor response group for achieving a TRG of 2–3. A comparison of pretreatment clinical parameters between the two groups is presented in Table 1. No significant variations were observed between the two groups regarding age, sex, body mass index (BMI), tumor location, tumor type, and chemotherapy treatments. Similarly, no significant differences were detected in the mismatch repair (MMR) status, KRAS status, and NRAS status between the two cohorts. However, there existed a few prominent differences between the two groups. The poor response group had a higher proportion of patients with advanced pretreatment clinical T stage and larger tumor length compared with the good response group (*p* < 0.001; *p* = 0.022, respectively). The presence of cT4 lesions was more frequently observed in the poor response group (49.5% vs. 22.6%). There was no discernible disparity in the clinical N stage between the two groups (*p* = 0.294). Furthermore, patients in the good response group exhibited a decreased percentage of positive extramural vascular invasion (EMVI) and circumferential resection margin (CRM), as assessed through MRI (39.8% vs. 53.1%, *p* = 0.035; 22.6% vs. 36.3%, *p* = 0.021, respectively).

**TABLE 1:** The pretreatment clinical parameters comparison between TRG0‐1 and TRG2‐3.

Factor	Good response *N* = 93	Poor response *N* = 190	*p* value
Age (years), mean (SD)	58.0 (12.9)	55.8 (11.8)	0.164
Gender, *n* (%)			
Male	35 (37.6)	72 (37.9)	0.996
Female	58 (62.4)	118 (62.1)	
BMI, mean (SD)	23.7 (3.1)	23.5 (3.2)	0.626
Distance from the anal verge (cm), mean (SD)	4.3 (1.9)	4.5 (2.0)	0.372
Gross type by colonoscopy, *n* (%)			
Ulcerative	24 (25.8)	70 (36.8)	0.077
Expanding	25 (26.9)	76 (40.0)	
Infiltrative	13 (14.0)	14 (7.4)	
Not reported	31 (33.3)	30 (15.8)	
Tumor length on MRI (cm), mean (SD)	3.4 (1.4)	3.8 (1.5)	0.022
Positive EMVI on MRI, *n* (%)	37 (39.8)	101 (53.1)	0.035
Positive CRM on MRI, *n* (%)	21 (22.6)	69 (36.3)	0.021
Pretreatment cT stage, *n* (%)			
cT2	15 (16.1)	5 (2.6)	<0.001
cT3	57 (62.3)	91 (47.9)	
cT4	21 (22.6)	94 (49.5)	
Pretreatment cN stage, *n* (%)			
cN0	38 (40.9)	65 (34.2)	0.294
cN+	55 (59.1)	125 (65.8)	
Initial CEA, *n* (%)			
≥5 ng/L	35 (37.6)	93 (48.9)	0.042
<5 ng/L	58 (62.4)	97 (51.1)	
Initial CA199, *n* (%)			
≥37 U/mL	12 (12.9)	39 (20.5)	0.139
<37 U/mL	81 (87.1)	151 (89.5)	
Initial AGR, mean (SD)	1.4 (0.2)	1.3 (0.2)	0.363
Initial PNI, mean (SD)	45.4 (6.3)	43.6 (6.7)	0.030
Initial NLR, mean (SD)	2.2 (1.3)	3.7 (6.7)	0.038
Initial SII, mean (SD)	612.0 (428.6)	808.2 (805.4)	0.029
Initial PLR, mean (SD)	135.4 (59.5)	147.7 (89.9)	0.170
T lymphocytes (cells/μL), mean (SD)	1470.8 (691.7)	1144.6 (728.9)	0.001
Th lymphocytes (cells/μL), mean (SD)	768.9 (432.0)	516.1 (318.5)	0.001
Tc lymphocytes (cells/μL), mean (SD)	418.9 (290.3)	440.3 (288.5)	0.559
Th/Tc ratio, mean (SD)	2.3 (1.5)	1.4 (1.2)	0.001
Natural killer cells, mean (SD)	181.2 (85.4)	165.0 (107.6)	0.206
Mismatch repair status, *n* (%)			
pMMR	73 (78.5)	128 (67.4)	0.081
dMMR	7 (7.5)	28 (14.7)	
Unknown	13 (14.0)	34 (17.9)	
KRAS status, *n* (%)			
Wild‐type	34 (36.5)	68 (35.8)	0.890
Mutation	46 (49.5)	97 (51.1)	
Unknown	13 (14.0)	25 (13.2)	
NRAS status, *n* (%)			
Wild‐type	46 (49.5)	76 (40.0)	0.103
Mutation	34 (36.5)	89 (46.8)	
Unknown	13 (14.0)	25 (13.2)	
Chemotherapy, *n* (%)			
Double‐agent	54 (58.1)	96 (50.5)	0.255
Single‐agent	39 (41.9)	94 (49.5)	

Abbreviations: AGR, albumin‐to‐globulin ratio; NLR, neutrophils to lymphocytes ratio; PLR, platelets to lymphocytes ratio; PNI, prognostic nutritional index; SII, systemic immune–inflammation index.

In terms of laboratory test parameters, carcinoembryonic antigen (CEA), PNI, NLR, SII, T lymphocytes, Th lymphocytes, and the Th/Tc ratio were related to the treatment response to NCRT in rectal MAC patients. Table 2 encapsulated the results of our multivariate logistic regression analysis, which revealed that tumor length (odds ratio [OR], 1.323; 95% confidence interval [CI], 1.086–1.621), pretreatment clinical T stage (OR, 2.751; 95% CI: 1.214–6.237), PNI (OR, 0.941; 95% CI: 0.897–0.987), and Th/Tc ratio (OR, 0.698; 95% CI: 0.534–0.941) were identified as independent risk factors for treatment response to NCRT.

**TABLE 2:** Independent predictors of poor response to NCRT on binary logistic regression.

Factors	OR (95% CL)	*p* value
Tumor length on MRI	1.323 (1.086–1.621)	0.005
Pretreatment cT stage	2.751 (1.214–6.237)	0.015
PNI	0.941 (0.897–0.987)	0.012
Th/Tc ratio	0.698 (0.534–0.941)	0.009

### 3.2 A nomogram predicting good treatment response

**FIGURE 1:**
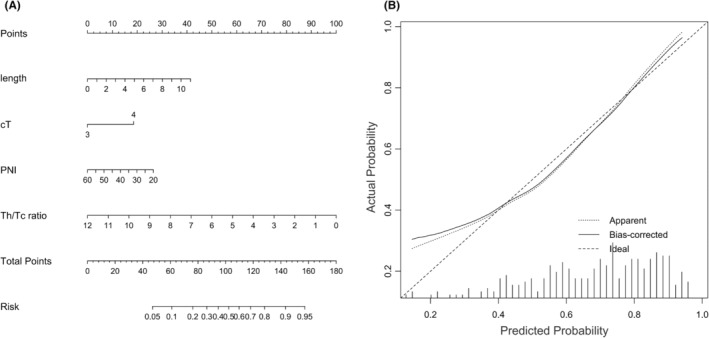
(A) A nomogram predicting the treatment response after neoadjuvant chemoradiotherapy. (B) Calibration curve for the nomogram depicts the fitness of the predictive events to the actual events.

We established a predicting nomogram to quantitatively visualize the contribution of each variable based on multivariate analysis. A greater overall score was associated with an increased likelihood of an unfavorable treatment response to NCRT (Figure 1A). The nomogram was internally validated and demonstrated a moderate predictive ability, with a C‐index of 0.756 (95% CI: 0.678–0.813). The analysis of calibration indicated a close resemblance between the predictive curve and the ideal curve (Figure 1B). By employing the most suitable threshold criteria, this model could effectively differentiate between a subpar response and a satisfactory response, achieving a sensitivity of 65.8% and a specificity of 75.3%.

### 3.3 AI model development and validation

**FIGURE 2:**
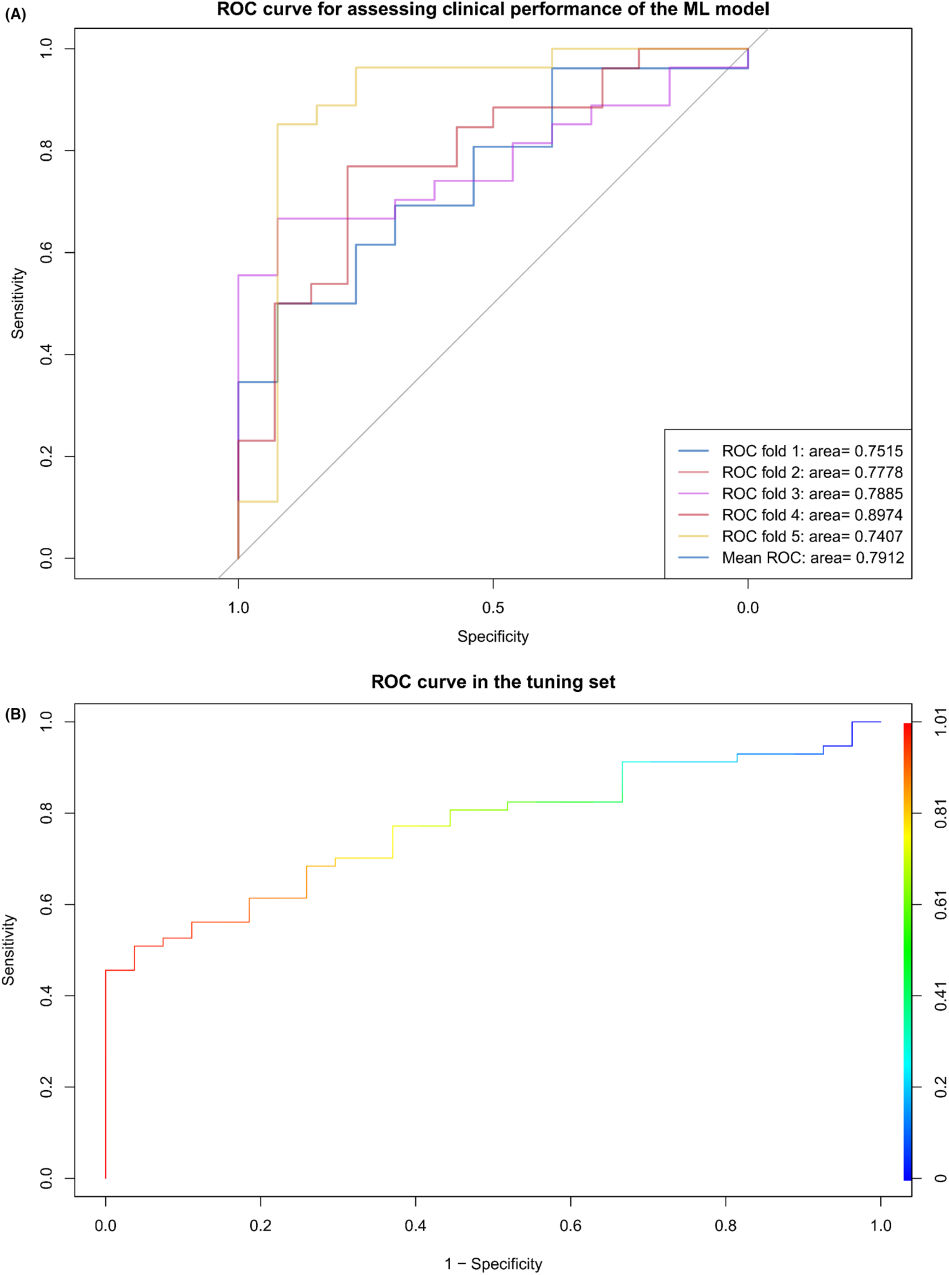
(A) ROC curve generated by five‐fold cross‐validation in the training set. (B) ROC curve in the tuning set.

**FIGURE 3:**
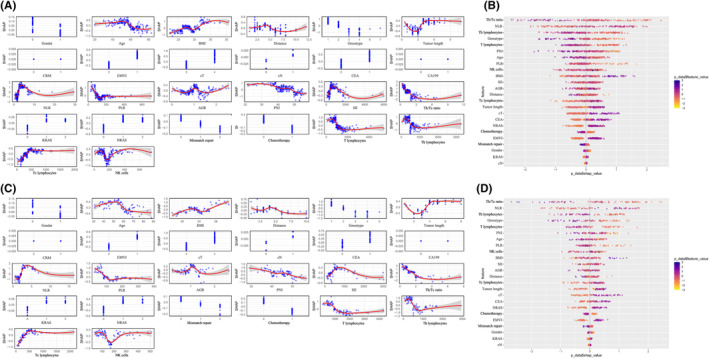
(A) Nonlinear distribution of each feature in the training set: the higher the absolute value of SHAP, the stronger the effect on the outcomes. (B) Feature importance rankings in the training set. The horizontal axis shows the relationship between features and tumor response probability, while the vertical axis displays variable names. Feature importance is determined by average SHAP values, with darker colors indicating stronger predictions. (C) Nonlinear distribution of each feature in the tuning set. (D) Feature importance rankings in the tuning set.

The clinical features of the training and internal validation datasets are specified in Supplemental Table S1. First, the training dataset was used to establish and train the ML model. To guarantee a well‐rounded and inclusive dataset, the AI model was initially run using its default hyperparameters and then fine‐tuned to attain an optimal model. The ROC curve generated by five‐fold cross‐validation in the training set and the ROC curve in the tuning set are depicted in Figure 2. The XGB model demonstrated strong performance in predicting treatment response in the training set, achieving an AUROC of 0.824, sensitivity of 67.6%, and specificity of 94.7%. Additionally, SHAP values were utilized to further elucidate the features that influenced this prediction model, after which the features were averaged to obtain their final importance ranks. We found that larger SHAP values indicated more significant contributions to influencing the response of the treatment to NCRT (Figure 3A). The Th/Tc ratio, NLR, Th lymphocytes, Gross type, and T lymphocytes ranked as the top five among all variables (Figure 3B). The AUROC in the tuning set was found to be 0.762, with a sensitivity of 58.2% and specificity of 89.5%, which closely matched the performance of the training set. Both sets demonstrated similar trends in evaluating feature importance.

## 4 DISCUSSION

The primary goal of NCRT for rectal cancer is to maximally resect the tumor, preserve the sphincter, and decrease the local recurrence rate.^12^ Surgeons often face an unresolved dilemma regarding whether to perform NCRT in rectal MACs. Patients diagnosed with rectal MACs who experience negligible TRG are unlikely to obtain benefit from NCRT, and there is a possibility that certain patients may even suffer adverse effects due to the postponement of definitive treatment and the chemoradiotherapy procedure.^13^ Preoperative prediction of tumor response can help mitigate the adverse effects and financial burden associated with chemoradiotherapy for patients with resistant tumors. Additionally, it allows for the consideration of more aggressive preoperative regimens for such patients. Herein, we have demonstrated the capability of circulating lymphocyte subsets and hematological inflammatory indexes in forecasting the response to treatment in patients with rectal MACs.

Based on earlier findings, approximately 40% of patients with rectal cancer do not respond to NCRT, with certain cases exhibiting disease progression while others showing a slight regression to stable disease.^14^ In our patient cohort, a higher proportion of patients (67.1%) were classified as exhibiting poor response (TRG 2–3). This suggests that the therapeutic effect of NCRT may be significantly reduced in MAC patients. However, the underlying mechanism of resistance to treatment is still unclear. Regarding the clinical characteristics, higher pretreatment clinical T stage and larger tumor length measured by MRI were identified as risk factors for poor treatment response, which was in line with previous studies.

At the molecular level, rectal MACs often exhibit various genetic aberrations and are significantly more likely to be associated with KRAS mutations, NRAS mutations, and dMMR.^15^, ^16^ In our patient cohort, the frequencies of KRAS mutation, NRAS mutation, and dMMR were 50.5%, 43.5%, and 12.4%, respectively. Furthermore, there was no significant correlation between the presence of KRAS and NRAS mutations and MMR status with the treatment response in MAC patients NCRT, possibly because immunotherapy or targeted treatments were not included in the preoperative regimen. Although the predictive impact of RAS oncogene and MMR status on the response to radiotherapy remains contentious,^17^ chemotherapy and immunotherapy choices are already influenced by RAS oncogene and MMR status. Emerging evidence suggests that the combination of NCRT with anticancer immunotherapy has demonstrated promising short‐term effectiveness in both pMMR and dMMR rectal cancers.^18^ Therefore, it is of utmost importance to conduct further research to ascertain whether the incorporation of anticancer immunotherapy can augment the effectiveness of NCRT in rectal MACs. A comprehensive understanding of the influence of immune indicators in peripheral blood on treatment response is essential for the meticulous design of immunotherapy protocols.

Inflammation is a well‐established risk factor for cancer development and progression. Moreover, the treatment response and oncological outcomes of rectal cancer are considered to be affected by the nutritional condition of individuals with cancer. Emerging predictors of the response to NCRT in rectal cancer include serological biomarkers related to inflammation and immunonutrition, such as SII, NLR, PLR, AGR, and PNI.^6^, ^19^ Our study revealed a significant correlation between the levels of SII, NLR, and PNI before treatment and the response to NCRT in patients with rectal MAC. Following the adjustment for possible confounding variables, the PNI level was identified as an independent indicator for predicting the response to NCRT treatment. The ML model ranked NLR as the second most important feature. This finding is consistent with most previous studies. One possible reason was that a decreased PNI or increased NLR could enhance the production of cytokines and inflammatory mediators, resulting in a weakened immune system and reduced local immune reaction, ultimately impacting the effectiveness of NCRT.^20^ However, the precise mechanism has yet to be ascertained. Further investigation is warranted to clarify the impact of anti‐inflammatory substances and immunomodulators on enhancing the treatment response in NCRT.

In addition to the hematological inflammatory markers, lymphocytes have a vital function in the systemic anticancer immune response induced by chemoradiotherapy. Numerous studies have focused on immune cell infiltration within the tumor microenvironment. The correlation between the density of Th lymphocytes infiltrating locally and the response to neoadjuvant therapy, as well as the prognosis of individuals diagnosed with rectal cancer, has been confirmed by multiple prior studies.^21^, ^22^, ^23^ Nevertheless, the effectiveness of NCRT cannot be predicted in advance based on the findings of research conducted on tumor‐infiltrating lymphocytes derived from postoperative pathological samples. At present, there is insufficient research investigating the association between different types of lymphocytes in the bloodstream and the TGR in NCRT for rectal MACs. Our study demonstrated that Th/Tc ratios, Th lymphocytes, and T lymphocytes were the top five important parameters of the ML predictive model. Additionally, the Th/Tc ratio was an important predictor in the nomogram model. These findings underlined the importance of circulating lymphocyte subsets in predicting treatment response in rectal MAC patients.

Currently, several studies have utilized inflammatory indexes or circulating lymphocyte subsets to predict treatment response in rectal cancers.^24^, ^25^, ^26^, ^27^, ^28^, ^29^ To our knowledge, so far, no study has reported the evaluation of the efficacy of NCRT for rectal MACs using the combination of inflammatory indexes and circulating lymphocyte subsets. In this study, we developed an XGB model based on preoperative clinicopathological parameters, inflammatory indexes, and the circulating lymphocyte subsets to predict treatment response to NCRT in rectal MAC patients. The XGB model demonstrates theoretical proficiency in predicting tumor response through the identification of intricate feature interactions, precise hyperparameter adjustments and mitigation of imbalanced datasets through oversampling and undersampling techniques. Empirical evidence supports the superior predictive performance of the XGB model over conventional linear models, as evidenced by AUROC values of 0.824 and 0.762 in the training and tuning sets, respectively, compared to an AUROC of 0.756 for the linear model. The top five variables associated with treatment response were identified as the Th/Tc ratio, NLR, Th lymphocytes, Gross type, and T lymphocytes. The XGB model demonstrated the ability to autonomously identify these factors similar to a nomogram, and also assisted in revealing the underlying patterns within the data for improved utilization of additional information. In contrast to nomograms, XGB's reliance on tree‐based models makes it more complex, requiring more computational resources and a substantial amount of data to prevent overfitting. Furthermore, the evolving nature of data distributions mandates regular updates and maintenance of ML models to uphold their predictive accuracy.

As inflammation indexes and the circulating lymphocytes can be accurately measured before treatment, the establishment of a predictive model based on these indicators may become a useful and cost‐effective approach to predicting treatment response. In the training set, our ML model exhibited a sensitivity and specificity of 67.6% and 94.8%, respectively, while in the tuning set, it demonstrated slightly reduced values with a sensitivity of 58.2% and specificity of 89.5%. Although the sensitivity of the ML model is slightly lower than that of linear prediction models (65.8%), the high specificity of the ML model implies that it has higher reliability and credibility in screening out patients who are unresponsive to NCRT.

It should be emphasized that the advanced clinical stage is a significant criterion for NCRT despite the clinical T stage being an independent risk factor for suboptimal response. However, the SHAP value of the clinical T stage did not rank high in our ML model (ranking 16). Therefore, when it comes to forecasting the efficiency of NCRT in individuals with advanced rectal MACs, employing an ML prediction model is more advantageous in clinical terms in contrast to employing a nomogram model. Consequently, we propose that patients with advanced rectal MACs who exhibit a potentially inadequate response to NCRT should be considered for an intensified treatment approach, such as the incorporation of targeted therapy or immunotherapy. Conversely, patients with locally early tumors and a potentially inadequate response to NCRT may benefit from direct surgical intervention. Moreover, providing patients with information regarding the probability of treatment response can assist clinicians in tailoring appropriate treatment strategies.

It is important to acknowledge several potential limitations inherent in our study. First, our analysis is limited by its retrospective nature, but it lays the groundwork for future prospective research. Second, we recognize the universal challenge of model interpretability associated with AI models. Third, the significance of circulating lymphocyte subsets in prognosis remains unclear. Therefore, validation and calibration through a large‐scale, multicenter trial are imperative. This trial should be structured as a longitudinal, prospective study, with a focus on evaluating the accuracy of the ML model. Subsequently, the utilization of ML models has the potential to aid in the assessment of the efficacy of NCRT for MAC. A prospective, randomized controlled trial could be conducted to allocate patients to either an ML‐supported intervention group or a conventional group. In instances where patients are expected to have suboptimal responses to NCRT, alternative approaches such as prompt surgical intervention or intensified chemotherapy protocols may be deemed appropriate. The primary outcome measure will be the disease‐free survival, with secondary outcome measures including local recurrence rates and overall survival.

## 5 CONCLUSION

In summary, our study demonstrated that the presence of systemic inflammation and immune reactions mediated by lymphocytes could markedly impact the sensitivity of NCRT in patients with rectal MACs. We constructed and verified an XGB model that incorporated various clinical factors, hematological inflammatory markers, and lymphocyte subsets. This model has demonstrated promising results in predicting treatment response. However, additional investigations are warranted to investigate the potential benefits of anti‐inflammatory agents and immunotherapy for enhancing the efficacy of NCRT in this patient population.

## AUTHOR CONTRIBUTIONS

**Yu Lin:** Writing – original draft (lead). **Weizhong Jiang:** Data curation (equal). **Yanwu Sun:** Software (equal). **Yu Deng:** Data curation (equal). **Ying Huang:** Writing – review and editing (equal). **Pan Chi:** Conceptualization (equal).

## FUNDING INFORMATION

This study was supported by Joint Funds for the innovation of Science and Technology, Fujian province (2023Y9131), Startup Fund for scientific research, Fujian Medical University (2020QH1065), Fujian Young Teacher Education Research Project (JAT200146), Joint Funds for the innovation of science and Technology, Fujian province (2019Y9065), National Clinical Key Specialty Construction Project (General Surgery) of China.

## CONFLICT OF INTEREST STATEMENT

The authors declare that there are no conflicts of interest.

## ETHICS STATEMENT

The Medical Ethics Committee of Fujian Medical University (FJMU) approved this study.

## CONSENT

All authors reviewed and approved the manuscript.

## Supporting information


Figure S1.

Table S1.

File S1.

## Data Availability

The data should not be publicly available because of ethical concerns.
