# Supplementary figures and images for: Predictive value of circulating lymphocyte subsets and inflammatory indexes for neoadjuvant chemoradiotherapy response in rectal mucinous adenocarcinoma patients: A machine learning approach
Source: Cancer Med. 2024 Jul 24;13(14):e7416. doi: 10.1002/cam4.7416 (PMC11267980; doi:10.1002/cam4.7416)

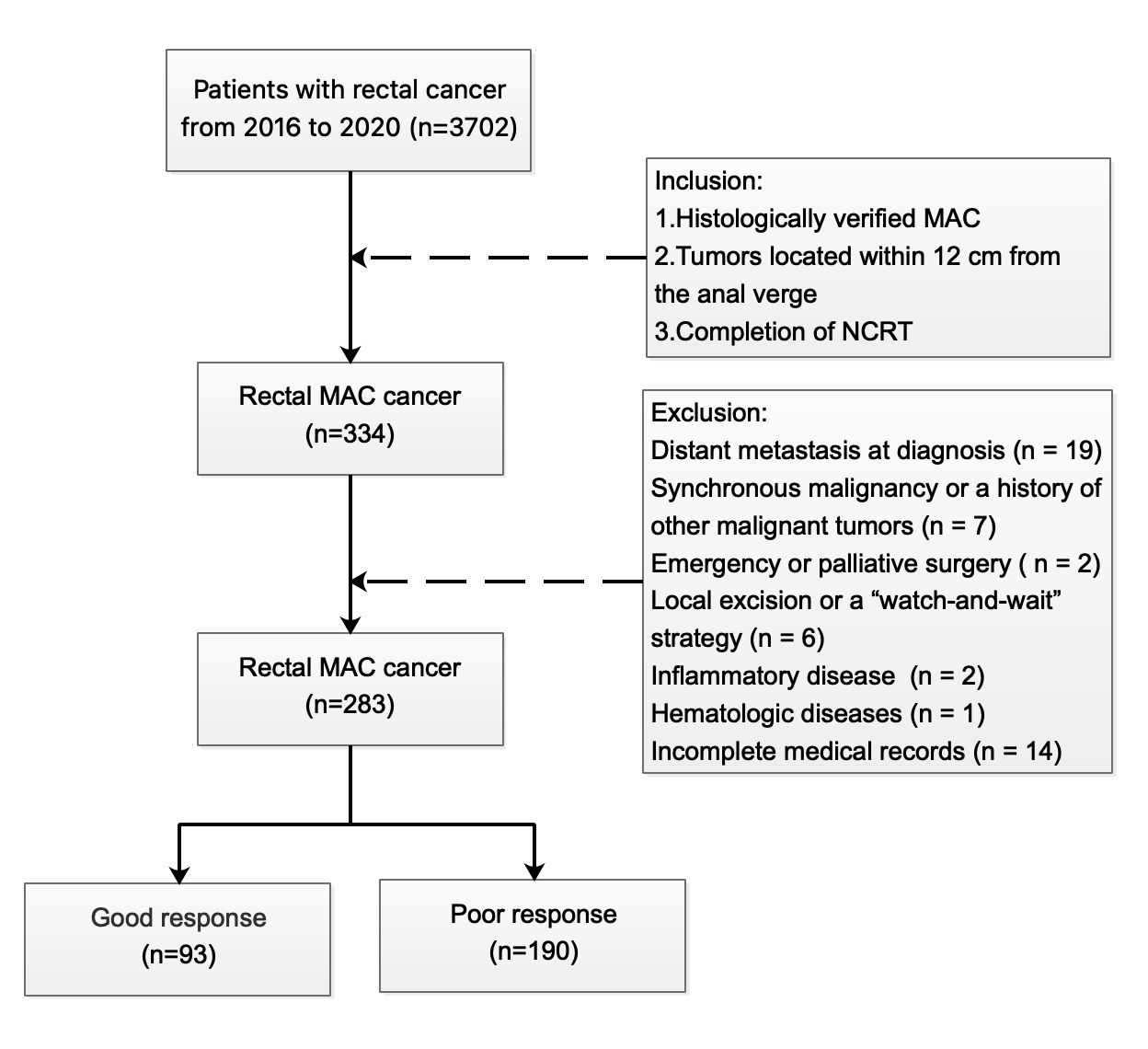

Supplement: Supplementary file 1 — Figure S1. [file CAM4-13-e7416-s001.tif]
