# Supplementary material for: Predictive value of circulating lymphocyte subsets and inflammatory indexes for neoadjuvant chemoradiotherapy response in rectal mucinous adenocarcinoma patients: A machine learning approach
Source: Cancer Med. 2024 Jul 24;13(14):e7416. doi: 10.1002/cam4.7416 (PMC11267980; doi:10.1002/cam4.7416)
Supplement: Supplementary file 2 — Table S1. [file CAM4-13-e7416-s003.docx]

Supplemental Table 1

The pretreatment clinical parameters comparison between training and tuning set

| Factor | All  N=283 | Training  N=198 | Tuning  N=85 | P value |
| --- | --- | --- | --- | --- |
| Age (years), mean (SD) | 56.6 (12.1) | 56.5 (12.5) | 56.6 (11.3) | 0.934 |
| Gender, n (%) |  |  |  | 0.444 |
| man | 107 (37.8) | 72 (36.3) | 35 (43.7) |  |
| woman | 176 (62.2) | 126 (63.7) | 50 (56.3) |  |
| BMI, mean (SD) | 23.6 (3.2) | 23.7 (3.2) | 23.3(3.2) | 0.377 |
| Distance from the anal verge (cm), mean (SD) | 4.4 (2.0) | 4.3 (2.0) | 4.6 (1.8) | 0.313 |
| Gross type by colonoscopy, n (%) |  |  |  | 0.213 |
| Ulcerative | 94 (33.2) | 72(36.4) | 22 (25.9) |  |
| Expanding | 101 (35.7) | 64 (32.3) | 37 (43.5) |  |
| Infiltrative | 27 (9.5) | 23 (11.6) | 4 (4.7) |  |
| Not reported | 61 (21.6) | 39 (19.7) | 22 (25.9) |  |
| Tumor length on MRI (cm), mean (SD) | 3.8 (1.6) | 3.9 (1.5) | 3.7 (1.9) | 0.373 |
| Positive EMVI on MRI, n (%) | 138 (48.8) | 92(46.5) | 46 (54.1) | 0.238 |
| Positive CRM on MRI, n (%) | 90 (31.8) | 63 (31.8) | 27 (31.8) | 0.993 |
| Pretreatment cT stage, n (%) |  |  |  | 0.239 |
| cT2 | 20 (7.1) | 12 (6.1) | 8 (9.4) |  |
| cT3 | 148 (52.3) | 110 (55.5) | 38 (44.7) |  |
| cT4 | 115 (40.6) | 76(38.4) | 39 (45.9) |  |
| Pretreatment cN stage, n (%) |  |  |  | 0.578 |
| cN0 | 103 (36.4) | 70(35.4) | 33 (38.8) |  |
| cN+ | 180 (63.6) | 128 (64.6) | 52 (61.2) |  |
| Initial CEA, n (%) |  |  |  | 0.198 |
| ≥5 ng/L | 130 (45.9) | 96 (48.5) | 34 (38.6) |  |
| <5 ng/L | 153 (54.1) | 102 (51.5) | 51 (61.4) |  |
| Initial CA199, n (%) |  |  |  | 0.434 |
| ≥37 U/mL | 51 (18.0) | 38 (12.9) | 13(20.5) |  |
| <37 U/mL | 232 (82.0) | 160 (87.1) | 72 (89.5) |  |
| Initial AGR, mean (SD) | 1.0 (0.47) | 1.0 (0.43) | 1.1 (0.54) | 0.235 |
| Initial PNI, mean (SD) | 44.2 (6.6) | 44.2 (6.5) | 44.1 (6.9) | 0.920 |
| Initial NLR, mean (SD) | 3.1 (2.8) | 3.2 (3.1) | 2.9 (1.6) | 0.393 |
| Initial SII, mean (SD) | 743.7 (709.3) | 730.0 (692.2) | 775.7 (751.0) | 0.621 |
| Initial PLR, mean (SD) | 143.7 (81.3) | 138.8 (70.3) | 155.1 (102.0) | 0.124 |
| T lymphocytes (cells/μl), mean (SD) | 1251.8 (732.0) | 1248.8 (688.9) | 1258.7 (828.0) | 0.918 |
| Th lymphocytes (cells/μl), mean (SD) | 599.1 (415.4) | 614.3 (406.4) | 563.7 (436.1) | 0.348 |
| Tc lymphocytes (cells/μl), mean (SD) | 433.3 (288.8) | 428.6 (291.2) | 444.2 (284.4) | 0.678 |
| Th/Tc ratio, mean (SD) | 1.7 (1.4) | 1.7 (1.3) | 1.7 (1.5) | 0.808 |
| Natural killer cells, mean (SD) | 170.3 (101.0) | 172.7 (90.2) | 164.7 (122.8) | 0.544 |
| Mismatch repair status, n (%) |  |  |  | 0.542 |
| pMMR | 201 (71.0) | 140 (70.7) | 61 (71.8) |  |
| dMMR | 35 (12.4) | 27 (13.6) | 8 (9.4) |  |
| Unknown | 47 (16.6) | 31 (15.7) | 16 (18.8) |  |
| KRAS status, n (%) |  |  |  | 0.412 |
| Wild-type | 102 (36.1) | 71 (35.9) | 31 (36.5) |  |
| Mutation | 143 (50.5) | 97 (49.0) | 46 (54.1) |  |
| Unknown | 38 (13.4) | 30 (15.1) | 8 (9.4) |  |
| NRAS status, n (%) |  |  |  | 0.429 |
| Wild-type | 122 (43.1) | 84 (42.4) | 38 (44.7) |  |
| Mutation | 123 (43.5) | 84 (42.4) | 39 (45.9) |  |
| Unknown | 38 (13.4) | 30 (15.2) | 8 (9.4) |  |
| Chemotherapy, n (%) |  |  |  | 0.989 |
| Double-agent | 150 (53.0) | 105 (53.0) | 45 (52.9) |  |
| Single-agent | 133 (47.0) | 93 (47.0) | 40 (47.1) |  |
